# Mental illness stigma’s reasons and determinants (MISReaD) among Singapore’s lay public – a qualitative inquiry

**DOI:** 10.1186/s12888-020-02823-6

**Published:** 2020-08-26

**Authors:** Gregory Tee Hng Tan, Shazana Shahwan, Chong Min Janrius Goh, Wei Jie Ong, Ker-Chiah Wei, Swapna Kamal Verma, Siow Ann Chong, Mythily Subramaniam

**Affiliations:** 1 Research Division, Institute of Mental Health, 10 Buangkok View, Singapore, 539747 Singapore; 2 Department of Developmental Psychiatry, Institute of Mental Health, Singapore, Singapore; 3 Department of Early Psychosis Intervention, Institute of Mental Health, Singapore, Singapore

**Keywords:** Social stigma, Mental disorders, Discrimination, Public attitudes

## Abstract

**Background:**

Mental illnesses pose a significant burden worldwide. Furthermore, the treatment gap for mental disorders is large. A contributor to this treatment gap is the perceived stigma towards mental illness. Besides impeding one’s help-seeking intentions, stigma also impairs persons with mental illness (PMI) in other aspects of their life. Studies have found that stigma may manifest differentially under different cultural contexts. Thus, this study seeks to elucidate the determinants of stigma towards PMI among lay public in Singapore using a qualitative approach.

**Methods:**

A total of 9 focus group discussions (FGD) were conducted with 63 participants consisting of lay public Singaporeans who were neither students or professionals in the mental health field, nor had they ever been diagnosed with a mental illness. Topics discussed during the FGD were related to the stigma of mental illness. Data collected were analyzed with inductive thematic analysis method. A codebook was derived through an iterative process, and data was coded by 4 different coders. Both coding and inter-rater analysis were performed with NVivo V.11.

**Results:**

In total, 11 themes for the determinants of stigma were identified and conceptualized into a socioecological model. The socioecological model comprised 4 levels of themes: 1) Individual level beliefs (fear towards PMI, perceiving PMI to be burdensome, dismissing mental illness as not a real condition), 2) Interpersonal influences (upbringing that instills stigma, intergroup bias, perceived inability to handle interactions with PMI), 3) Local cultural values (elitist mindset among Singaporeans, Chinese culture of “face”, Islamic beliefs about spiritual possession and reaction towards PMI), and 4) Shared societal culture (negative portrayal by media, Asian values).

**Conclusions:**

The findings of this study improved our understanding of the various reasons why stigma exists in Singapore. The themes identified in this study concur with that of studies conducted overseas, as some determinants of stigma such as fear towards PMI are quite ubiquitous. Specifically, the themes elitist mindset among Singaporeans and perceived inability to handle interactions with PMI were unique to this study. It is highly recommended that future anti-stigma campaigns in Singapore should incorporate the findings of this study to ensure cultural misgivings and beliefs are addressed adequately.

## Background

Mental illnesses pose a significant burden worldwide. Data suggests that more than a billion people are affected by mental illness globally, with these individuals accounting for 7% of all global burden of disease measured in disability-adjusted life year (DALY), and 19% in terms of all years lived with disability [1]. However, despite robust evidence showing the efficacy of treatment and psychological therapies for common mental disorders such as depression and anxiety [2, 3], there still exists a wide treatment gap [4]. Studies have evinced that individuals with untreated mental illnesses have increased contact with the criminal justice system, are less likely to be employed, and more likely to die prematurely [5–7]. At a societal level, untreated mental illness can lead to reduced productivity, as well as greater spending on healthcare and other welfare expenditures [8, 9].

There are several factors that contribute to the wide treatment gap for mental illness, with stigma being acknowledged as a significant reason. Stigma affects an individual’s help-seeking intentions and behaviors, insomuch that a person considering treatment may be discouraged from doing so due to the anticipation of potential discrimination. Such beliefs may arise from the stigma that is attached to help-seeking, as they may fear being labelled with a diagnosis that puts them in the stigmatized group (label-avoidance) [10]. Besides impeding an individual’s help-seeking intentions, stigma also impairs an individual’s life in other aspects. As a result of stigma, a PMI may be denied equal life opportunities such as employment and housing, discriminated by the justice system, and suboptimal treatment by the general healthcare system [11]. Other social impacts of stigma include social distancing and unwillingness to engage in close relationships with PMI. Perhaps more troubling is the internalization of society’s stigmatizing notions, which undermines the self-esteem and sense of self-worth of PMI, which may in turn, worsen their mental health [12]. In fact, many individuals with mental illness have proclaimed that the experience of mental illness stigma may be worse than the condition itself [13].

The stigma of mental illness is one that is ubiquitous and can be considered universal [14]. This is supported by Thornicroft et al.’s review, which found that the experience of stigma and discrimination is common [15]. A cross-sectional survey conducted across 35 countries of individuals with a diagnosis of major depressive disorder also generated similar findings, where 79% of respondents reported experiencing discrimination, such as being treated unfairly by people in their neighbourhood or challenges in finding a job by virtue of being a PMI [16]. Although stigma may constitute a universal phenomenon, stigma may manifest itself in different ways under different cultural contexts and settings [14]. A case in point would be the study by Shamblaw et al. (2015), which found that Asians were associated with greater stigma towards mental illness than Europeans, and the belief that depression carries familial shame was cited as a mediator between ethnicity and stigma [17].

Singapore is a multi-ethnic nation in Southeast Asia whose citizens are comprised of mostly Chinese (74.4%), followed by Malays (9.0%) who are predominantly Islamic followers [18], Indians (9.0%) and others (3.2%) [19]. An epidemiological study in Singapore found considerable stigma towards people with mental illness [20]. Although majority of participants in this study expressed willingness to spend an evening or be friends with a PMI, a substantial percentage of them expressed unwillingness to move next door to a PMI (32.4%) and to work closely with a PMI (42.8%). Additionally, most expressed that they would not be willing to have a PMI marry into their family (70.2%). Also warranting concern was that majority of participants felt that PMI could get better if they wanted to (89.4), that PMI were unpredictable (62.5%), and that the illness was a sign of personal weakness (50.8%).

In Chinese societies, stigma towards mental illness is intertwined with their cultural norm of “face”, a construct that is central to one’s social identity, representing one’s power and standing in the social hierarchy among Chinese. Thus, an onset of mental illness usually brings about a “loss of face” for both the individual and the associated family members, because of the pejorative views on the etiological beliefs of the illness, such as a moral “defect” among the sufferer’s family [21]. Some other etiological beliefs of mental illness among Chinese societies that might lead to stigma includes; the belief that mental illness is caused by the bite of a rabid dog (which possibly instills fear of transmission) [22]; a misconduct by one’s ancestors resulting in punishment in the form of mental illness [23]; and the beliefs about mental illness being hereditary in nature which implicates the family as being pathogenic [24].

The etiological beliefs of mental illness among Indian culture are seemingly different from that of Chinese societies, and likely to influence stigma differentially as well. A study in South India found that among several non-biomedical etiological beliefs of mental illness such as karma, black magic, punishment by God and evil spirits, stigma was associated only with the belief that mental illnesses are caused by karma and evil spirits [25]. Another study in North India revealed that majority of patients had undergone ‘magico-religious’ treatment for their illness, which was likely attributable to the belief that their symptoms were caused by supernatural influences [26]. A more recent study in Delhi revealed that although participants did endorse biomedical explanations for causes of mental illness, there were still considerable endorsements of non-biomedical causes such as “God punishing for past sins”, “witchcraft” and “loss of semen/vaginal secretion” [27].

Likewise, followers of Islam are susceptible to the misperceptions that mental illness arises from ‘supernatural activities’, as revealed by a study in Malaysia [28]. This finding is consistent with an earlier study that elucidated that Malay Muslims are likely to attribute the cause of psychiatric illness to fate and religion [29]. The attribution of mental illness to supernatural causes is prevalent in Indonesia as well, and PMI often have to cope with being accused of not being religious enough to fight off evil spirits and thoughts [30]. However, there were mixed findings with regard to the reaction towards mental illness arising from such etiological beliefs. Though such beliefs can elicit disdainful reactions towards PMI, Muslims tend to appraise themselves as being “responsible for people with mental health issues”, and failing to do so is essentially viewed as “defying God’s will”, since mental illness is regarded as a trial by God to test their faith [28, 30]. These findings illustrate the importance of studying stigma in various cultural contexts in order to gain a better understanding of the cultural influences of stigma.

According to Kutcher et al., “Mental health literacy interventions need to be contextually developed and developmentally appropriate”, and such interventions have to be framed in appropriate domains and delivered in the context relevant to the target audience [31]. Although there has been earlier work published pertaining to stigma in Singapore, none of those were qualitative in nature [32, 33]. Therefore, this study seeks to elucidate the determinants of the stigma towards mental illness in Singapore and to investigate the cultural influences of stigma if any, from the perspective of the general public, by utilizing a qualitative approach.

## Methods

This qualitative study was part of a bigger study that aimed to examine the nature of mental illness stigma in Singapore from the perspectives of five stakeholder groups, namely the general public, PMI, caregivers of PMI, professionals in mental health setting, and policy makers. The study was approved by the National Healthcare Group Domain Specific Review Board. Written informed consent was obtained from all participants before initiating study related procedures. Each Focus Group Discussion (FGD) lasted between 1.5-2 h, was audiotaped and transcribed verbatim by a member of the study team.

### Sample

Members of the public were recruited through convenience and snowball sampling. The study was advertised to the public by word of mouth and brochures. Brochures were given to participants and to the friends of study team’s member to help advertise the study to potential participants. Interested participants would then pass their contact details to the study team. The study team would then coordinate with the participants and arrange for a FGD. Inclusion criteria comprised 1) being 21 years and older; 2) able to converse in English as all the FGDs were conducted in English; 3) not a student or professional from the mental health field; and 4) not been diagnosed with a mental illness (as there was a separate study for PMI).

### Data collection

A topic guide comprising open-ended questions (refer to Table 1) was developed by the research team to make sure that data collected across the various FGDs would be as uniform as possible. Each FGD was conducted by two team members, a facilitator and a note-taker. Written informed consent was taken and the participants’ socio-demographic information was also collected. Prior to the start of the discussion, participants were assured that data collected would be kept confidential and anonymized, and that there were no right or wrong answers/opinions.

**Table 1:** FGD Topic List

**Thoughts about Mental Illness and Stigma** -Can you describe a PMI? What comes to mind?	
-Can you tell me more about such (repeat terms participants used to reflect stigmatizing attitude to first question if any) that you or other people have towards mental illness?	
**Follow-up questions on vignette (30mins)**	
-What, according to you, would people think about this person?	
-Can you describe some of the positive or negative perceptions they might have?	
-Do you think people will be willing to work with the person described in the vignette?	
-Do you think people will be willing to include this person in their social or friend circle?	
**Role of culture in stigma**	
-Some people believe that the culture of the society plays a role in stigma. Would you agree with that? Can you tell us why you think that way?	

The first few questions in the guide (refer to Table 1) were meant to elicit responses from participants on their thoughts about mental illness, and to inquire more about their perspectives on the reasons for stigma. Next, participants were handed a vignette describing a male with symptoms of either depression or schizophrenia, depending on whether the FGDs were assigned either the depression or schizophrenia vignette (refer to supplementary file 1). Once participants had read the vignette, a series of questions were asked pertaining to the vignette to evaluate participants’ recognition of mental illness, and their opinions of a person exhibiting symptoms of mental illness. These were followed by questions which probed the participants on whether they would be willing to work with and/or be socially inclusive towards person described in vignette.

Lastly, participants were asked to opine on whether culture plays any role towards the stigmatization of individuals with mental illness (refer to Table 1). Data collection ended after 9 FGDs as no new information surfaced, indicating that data saturation was reached.

Besides facilitating the discussion, the facilitator also clarified any comments that were inconsistent, vague or ambiguous, and provided a summary of the FGD content at the end of each section, while the note-taker took careful notes of the entire discussion and seating arrangement. The other responsibility of the note-taker was to note down if any specific group members (based on age, ethnicity, gender etc) held notably different views or had been reluctant to express their views. Each session was facilitated by either Mythily Subramniam (MS) or Shazana Shahwan (SS), both of whom are trained and experienced in qualitative research methodologies, while the notetaking was assigned to either of the other 3 members of the study team, namely Chong Min Janrius Goh (CMJG), Gregory Tee Hng Tan (GTHT) and Wei Jie Ong (WJO). Immediately at the end of each FGD, there was a debrief between the facilitator and note-taker and later with the rest of the research team to ensure that problems were identified early and emerging themes were discussed in terms of overlap with other sessions or their unique associations with a particular ethnic group.

### Analysis

Transcripts of FGDs were checked through by facilitators to ensure accuracy, and data were analyzed with the inductive thematic analysis method [34], where content was coded to identify emerging themes. Each of the study team members independently conducted a preliminary analysis of a subset of FGD transcript, and the open coding method was used to identify and generate key themes [35]. Keeping the original questions in mind, study team members familiarized themselves with the data, and quotes of relevance in the transcript were highlighted, alongside recording of preliminary thoughts about the highlighted quotes. The study team then discussed and reviewed the highlighted quotes and sorted the quotes with similar content into piles of quotes that were representative of an abstract concept. The themes were then generated via an iterative process of grouping concepts into themes based on their common properties, examining quotes within each theme for congruency with the theme, and refining of the themes to ensure minimal overlap between themes that were meant to be discrete.

Consensus for any disagreements were reached through discussions and iterative review of the codes and themes, and a codebook was developed. All the study team members (except MS) coded the same two transcripts using the codebook developed to establish inter-rater scores. Upon achieving a satisfactory Kappa score, the other transcripts were then disseminated to GTHT, CMJG, WJO and SS for independent coding. The established Kappa among the 4 team members ranged from 0.78–0.93, which can be interpreted as substantial according to Cohen’s suggestion, where 0.61–0.80 represents substantial, and 0.81–1.00 symbolizes almost perfect agreement [36]. All analysis and inter-rater reliability tests were performed using Nvivo V.10. (QSR International. NVivo V.10 Computer software).

## Results

**Fig. 1:**
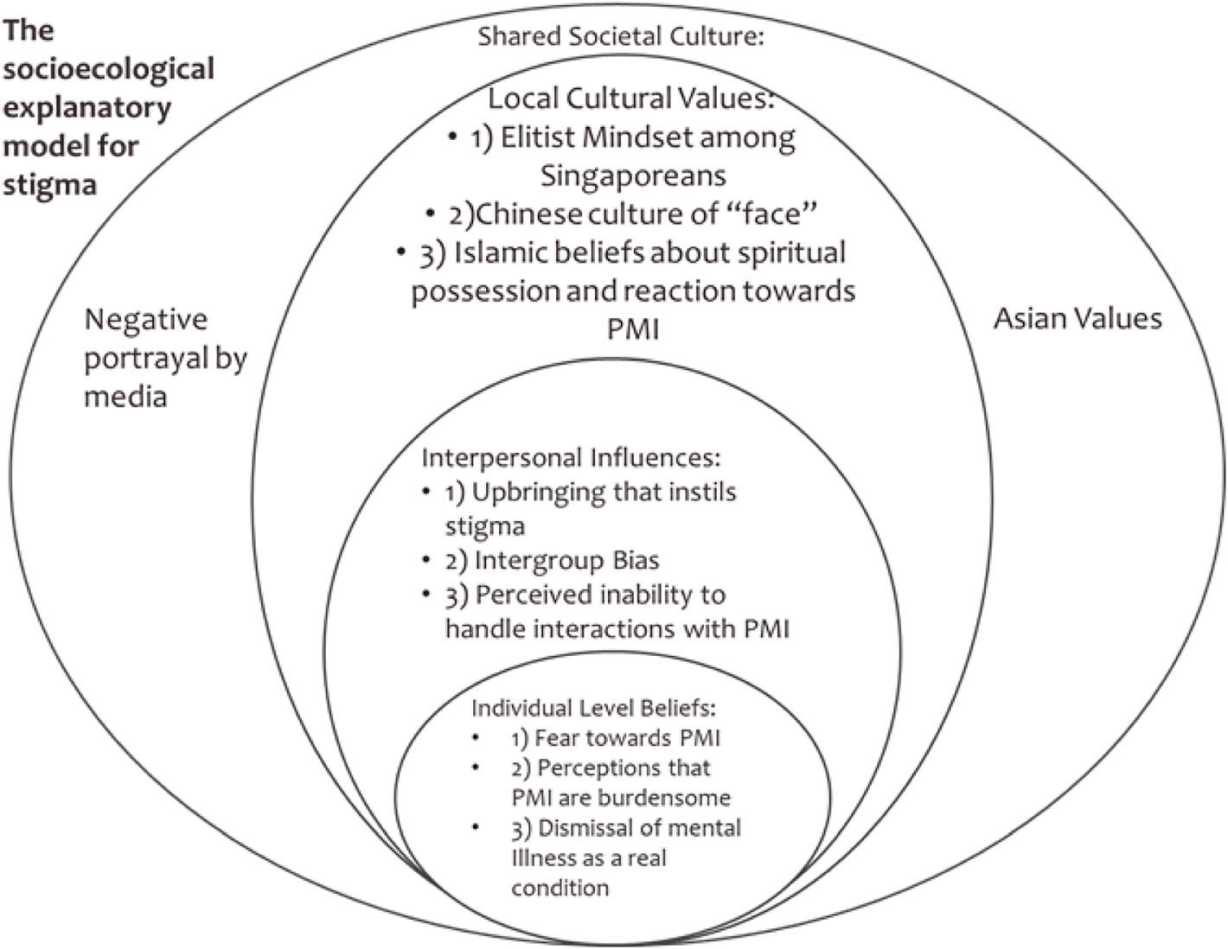
Socioecological Model of Stigma in Singapore

A total of 62 participants between the ages of 21 to 60 (mean = 33.5 ± 12.1) were recruited over 9 FGDs with nearly half of all participants being males (*n* = 30). Refer to Table 2 for participants’ sociodemographic information. In all, 11 broad themes emerged regarding reasons for stigma towards PMI. Drawing on Baral et al.’s modified socioecological model [37], we classified the themes into 4 broad overarching themes (levels) and proposed our own socioecological explanatory model for stigma (SEMS) amongst the general public in Singapore (refer to Fig. 1). The four levels of the model, in ascending wideness of socioecological network are Individual Level Beliefs (person’s internalized beliefs about PMI which drives stigma), Interpersonal Influences (stigmatizing beliefs about PMI which are possibly influenced by social interactions), Local Cultural Values (stigma towards PMI which are fostered by local culture) and Shared Societal Culture (encompasses culture beyond the local that fosters stigma). To ensure that standard usage of English is maintained, minimally corrected verbatim of quotes are presented. A table was also included to show the endorsement of each themes by FGD units (see Table 3).

**Table 2:** Sociodemographic Characteristics

	Mean	S.D
Age	33.5	12.1
	n	%
Sex		
Male	30	48.4
Female	32	51.6
Minimum Education Completed		
Secondary and Below	11	17.7
Vocational Institutional Education/Diploma/Pre-U	23	37.1
University Degree and above	28	45.2
Ethnicity		
Chinese	24	38.7
Malay	19	30.6
Indian	15	24.2
Eurasian and Others	4	6.4
Religion		
Christianity and Catholic	12	19.4
Buddhism and Taoism	8	12.9
Islam	22	35.5
Hinduism	9	14.5
Agnostic and Atheist	11	17.7
Marital Status		
Married	22	35.5
Single or Not Married	40	64.5

**Table 3:** Endorsement of themes among the 9 FGD groups

Themes	Endorsed By FGD Group(s)	Total Units
Fear towards PMI	1–9	9
Perception that PMI are burdensome	1,2,4,5,6,8	6
Dismissal of mental illness	1,2,3,7,8	5
Stigmatizing upbringing	1,2,3,6,7,8	6
In-group vs out-group	1,2,3,4,8,9	6
Perceived inability to handle interactions with PMI	1,2,3,4,5,6,7	7
Elitism mindset among Singaporeans	1,2,4,8	4
Chinese culture and “face”	1,3,4,5,8	5
Attributing MI to spiritual possession	1,2,5,6,7,9	6
Negative portrayal of MI by media	1,2,3,4,5,9	9
Asian conservative and collectivist values	1,2,4,6,7	5

### Individual level beliefs

#### Fear towards PMI

Participants’ responses indicated some level of fear towards PMI and as a result they expressed aversion towards them. The perception that PMI are dangerous came across as the most significant explanation for lay public’s fear and aversion towards PMI

"Sometimes, fear can also come in the fact of maybe it can be dangerous being beside this person."– [Age: 45]

In addition to the fear of violence, participants also talked about a fear of the unknown – feeling of fear that is triggered by a perceived lack of information at any level of consciousness [38], which plausibly stems from a lack of understanding towards PMI and the perception that they are unpredictable.

"I mean for me; I think every human we are fearful of the unknown. So like when we don’t know stuff, we tend to be more wary while approaching that fear thing." [Age: 21]

Lastly, hygiene-related reasons were also cited as to why a person would avoid being in close-proximity with a PMI

"Normally people tend to stay away from them especially they don’t groom themselves; they start to smell bad and all." [Age: 43]

#### Perception that PMI are burdensome

Participants had a perception that PMI are incompetent at work and burdensome to society and this contributed to the stigma in two ways. Firstly, there was a greater unwillingness to work with a PMI (versus someone without a mental illness) as they were considered incapable of carrying their own weight when it came to work.

"if it is my colleague and we are part of a project then, then I will feel that…assuming that our bonus is at stake etc, then I might feel that he might be a…he might drag the team down and that is my honest opinion." [Age: 23]

Secondly, PMI were also seen as burdensome to society in other ways such as requiring greater resources (participants did not explicitly mention whether this refers to resources from family or from society in general) such as money, time spent or effort being dedicated to them despite them not making any meaningful contribution to the society.

"I think most importantly is the financial cost, like the amount of money putting into…trying to rehabilitate them. And also, the loss in terms of what they could actually contribute to the society. That’s the burden." [Age: 23]

#### Dismissive towards mental illness being a real medical condition

The repudiation towards mental illness being a legitimate medical condition appeared several times during the discussion where some participants subscribed to the weak-not-sick beliefs [39] about mental illness.

"say for example depression or mental illness, the first thing that people think about you is that you are just not resilient. “Everybody goes through hardship, wake up your idea and get on with life." [Age: 43]

Some also expressed the opinion that PMI were just using mental illness as an excuse for their shortcomings or to circumvent their way out of their responsibilities.

"the word depression gets thrown around very loosely nowadays you know, I can say I am just…a lot of people just say I am depressed very easily, so this kind of thing I am afraid it becomes a habit…erm you…actually there’s nothing wrong with you but you are treating something that is not there" [Age: 24].

### Interpersonal influences

#### Upbringing that instils stigma

Prior research on prejudice has found that ethnic prejudice can be fostered due to upbringing [40, 41]. Consistent with those studies, we found that stigmatizing views towards mental illness can also be passed down in one’s family due to upbringing by the older generation which instilled certain negative preconceived sentiments about PMI such as the notion that PMI are dangerous.

"it really depends on what is your family upbringing of you towards mental health. Yah, probably your parents might say ‘this person is siao (a pejorative term in Hokkien which means crazy) one, don’t go near, later you will get killed or you get stabbed". [Age: 25]

Participants cited a lack of mental health literacy (MHL) on the part of the older generations as the reason for why this occurred.

"maybe our past generation or whatever, they don’t have any understanding of mental illness and then they pass it to their children or all of these. Actually, I went through a supermarket, I think it was last weekend. An old lady was like "oh don’t get so close", was telling the children not to get too close to the person because the person is siao" [Age: 25]

#### Intergroup bias

Intergroup bias also surfaced as one of the reasons for stigma in the study, with PMI recognized as the out-group. Participants mentioned that stigma towards PMI may arise because of the general out-group discrimination towards people who are different.

"Then a simpler way to look at them being stigmatized is because people with mental issues they’re different. So I feel that we… because you know when you see someone else acting differently like not a normal person, people will just go “this one not normal, this one crazy." [Age: 23]

Further, participants also mentioned concerns about associative stigma which leads to an avoidance of PMI.

"I think because he is mentally crazy so being friends with someone like this sort of put in like a… I don’t know how to put it, but in a more negative light in front of other people." [Age: 23]

#### Perceived inability to handle interactions with PMI

This theme describes participant’s perspectives on why they felt that they would rather not be in close contact with a PMI and consists of 3 sub-themes, and such beliefs possibly leads to greater social exclusion of PMI in the society. The three sub-themes included a perceived lack of expertise to deal with PMI (characterized by a feeling of a lack of adequate knowledge or understanding on how to interact with a PMI),

"I think because dealing with them right, sometimes we are not trained and we don’t know how to deal with them. So like maybe we try to help someone with mental illness and it didn’t work out, then we just give up because we try and it make them feel worse you know." [Age: 22]

perceived lack of resources to deal with PMI (characterized by one’s supposition that having contact with PMI will result in a lot of hassle and burden on themselves),

"we just try to live life with less problem so if I put myself in there, I am going to make that person’s problems my problems. So like I might have to talk to…take care of them that kind of thing. Example will be I might come in for questioning, so I already have other things that I needed to do and not have any more extra problems added on." [Age: 27]

and the perceived inability to ward off PMI’s negativity i.e., that their low moods or negativity would affect the participants well-being.

“you wouldn’t want to be with someone who has negative vibes around. Yea, then you also being pulled into that negative vibe and you don’t feel good about yourself. That’s why people tend to avoid it” [Age: 23]

### Local cultural values

#### Elitist mindset among Singaporeans

Several of the participants remarked that in Singapore, there is an expectation that one has to be successful/productive, and alluded that PMI tend to be ostracized or looked upon with contempt because of their failure to conform to these high standards.

“A person with mental illness requires care, he is not independent and therefore he is deemed as a loser and you don’t…the society doesn’t allow you to be associated with because it means you are a loser too. So I think it is because of this culture of “No, you have to be successful,” “you have to be independent to be…for people to want to believe you.” That’s probably one of the main reasons why people tend to shun away from people with mental illness because they have perceived the people who are losers that can’t take care of themselves.” [Age: 43]

#### Chinese culture and “face”

Several of the Chinese participants who shared their input on how culture affects stigma stated that mental illness would lead to shame and loss of respect for one’s family, which are related to the Chinese cultural construct known as “face”, a construct that is deeply intertwined with social standing [21].

“For Chinese I think it is more of the face, like how much less respect you get if someone in your family is known to have mental illness.” [Age: 26]

In this case, the stigma towards mental illness may manifest in the form of denial or the refusal to acknowledge one’s condition, due to the perceived repercussion of so doing, such as the loss of social standing should others find out that there is a PMI within the family.

“for the Chinese side and the Singaporean culture on the whole, I think as a Chinese in Chinese culture, we very hush-hush you know, we don’t want to lose face in the sense…I mean when, when you say you have something wrong or when you, when you show your shortcomings, it feels like people will look down on you, so if I say that I have mental illness, people will be automatically think something else of me, that’s why there is a perfect, we try and show we are perfect in a way.” [Age: 24]

#### Islamic beliefs about spiritual possession and reactions towards MI

Although the attribution of mental illness to spiritual possession is not unique to Islam alone, our findings suggest that this belief was more prevalent among the Muslims in Singapore

“To me in our society, we are Malay, maybe Malay Muslim, like what I know from my aunties and uncles, they always think these kind of people are possessed.” [Age: 32]

At the same time, some participants remarked that the Islamic culture is more accepting towards mental illness and PMI, although the motivations behind such prosocial behavior was not elaborated.

“Another thing is that if you see the Malay community is more together and they also accept every if there is a mental, they know…the whole family knows about this but they still accept it in a way.” [Age: 54]

### Shared societal culture

#### Negative portrayal of PMI by media

Congruent to the existing literature that evinced the relationship between negative media portrayal of mental illness and stigma [42, 43], our findings revealed that participants were exposed to stigmatizing portrayals of mental illness, where mental illness is associated with violence, which led to them endorsing beliefs that PMI are dangerous.

“The 9PM shows, and then usually the bad person in the movie, maybe something happened to him or her and then suddenly become depressed, and it always ends up either killing or injuring someone. So, when I grew up, my impression is like when someone is depressed, confirm will turn violent.” [Age: 21]

#### Asian conservatism and collectivism

Several participants intimated that the stigma towards mental illness is harsher in Singapore compared to the Western world, stating that Asians are more conservative while Westerners are more liberal. For instance, one participant made a comparison of Asian culture with the United States where people are more open about mental illness

“Like for example in US right, the culture of therapy, seeing a therapist is actually very common and therapists are celebrated. You know, even normal people go to therapy.” [Age: 33]

Another participant spoke about how Asians are very “closed off” whereas those from Western countries are more outspoken.

“It’s definitely the Asian culture because we, however we are in Singapore, maybe we don’t have to look far, it’s just the Asians that we are, we are very closed off. Maybe if we compare ourselves to like the Westerns, they are very outspoken like they talk about these kind of things.” [Age: 26]

One participant also commented about how the Asian collectivist culture can contribute to in-out group stigma.

“I think that the Asian culture does play a strong role in stigma? It also has a very bad footing when it comes to someone who is outside that regular group.” [Age: 31]

### Non-stigmatizing views of PMI

We have also included some comments made by participants, although not many, that are non-stigmatizing to provide a contrast. Participants commented that PMI can be just like any other normal people, and be as productive as well.

“They are highly functional, very bright individuals. The only difference in fact is like if they don’t come out or tell you or if you are not trained in the profession, you wouldn’t have known that they do suffer from certain level, certain degree of mental illness” [Age: 43]

One participant even remarked that PMI can be the ones to be spreading positivity, a contrast from our third theme that PMI are contagiously negative.

“The positive thing about it is that people with mental illnesses, they want people around them to be happier than what they are. So they do more to make the people around them happy so they sort of feel happier themselves as well.” [Age: 22]

## Discussion

Our findings unearthed the various determinants of stigma among the lay public in Singapore. In all, 11 themes were observed. Drawing from Baral et al.’s work [37], these themes were categorized into different levels to better conceptualize a model that elucidates the determinants of stigma among the general public in Singapore (see Fig. 1). The lowest level in the model represents the internalized beliefs that an individual has which incites stigmatizing attitudes towards PMI. The themes comprising the next higher level in the model are determinants of stigma which are motivated by the individual’s interactions with others, followed by the local cultural level stigma determinants, and the highest level encapsulates cultural determinants of stigma that are more global in their representation.

The boundaries between the various levels in our model are porous such that determinants of stigma can have bi-directional influences. For instance, stigma may be inculcated when an individual who had an “Upbringing that instills stigma” (Interpersonal influences) internalizes those beliefs and end up fearing PMI (Individual level beliefs). As one participant puts it, it is due to the upbringing which instills stigma that people are taught to perceive PMI as dangerous (see quote in results section: 1. Upbringing that instils stigma). At the same time, upbringing can also instill local cultural values - one level above Interpersonal Influences -such as “face” which may propagate stigmatizing outlook towards mental illness. Of course, it is also possible for stigma determinants to directly influence levels further than one level apart. A case in point, “elitist mindset among Singaporeans” and the perception that PMI are “burdensome to society” may be interlinked, insomuch that one might be more disposed to view PMI as burdensome if they possess an elitist mindset.

There are several generic causes of mental illness stigma in our study which are similar to those reported in studies conducted in other countries. For instance, the relationship between fear and social distance towards PMI has been documented by many studies [44]. Link et al.’s study conducted in Ohio found social distance to be correlated with participant’s perceived dangerousness of the mentally ill patient [45], and a review of stigma towards mental illness in Asia revealed similar findings to Link et al.’s study [46]. The notion that PMI are incompetent at work and burdensome is pervasive among the general public in Europe, as evinced by a review [47], and this surfaced in our findings as well. Other determinants of stigma that our study has identified such as upbringing [40, 41], intergroup bias and concerns about associative stigma [48, 49], and the perception that PMI are “weak-not-sick” [39] were also similar to earlier findings from overseas studies. Hence, this suggests that there are some causes of mental illness stigma which are quite pervasive globally.

In contrast, the sub-theme perceived inability to handle interactions with PMI is more unique to our study. We postulate that an individual’s perceived inability to handle interactions with PMI may be attributed to a lack of knowledge and/or awareness about mental illness in general (perceived lack of expertise) or previous unpleasant experiences of interacting with PMI which evoked feelings of inadequate self-efficacy in socializing with PMI (perceived inability ward off PMI’s negativity). Lastly, participants’ perceived lack of resources to deal with PMI may be attributable to Singapore being a relatively fast-paced society. This hypothesis is inspired by Levine and Norenzayan’s work (1999) on the pace of life in 31 countries, which suggested that people in cities with a faster pace of life were less likely to engage in prosocial behavior, as they have less time to devote to factors peripheral to their main goals [50].

Our study also identified a few themes which shed light on how culture possibly influences stigma. One aspect of culture that influenced stigma in Singapore is the Chinese construct of ‘face’ and its societal importance. There is a widespread belief denoted by the Chinese culture that mental illness is a result of some form of moral “defect” on the sufferer’s part or within their family. This possibly explains why earlier local studies found Chinese to have less social tolerance and more negative attitudes towards PMI as compared to other ethnicities [32, 33]. In any case, the implications of such cultural beliefs probably resulted in mental illness being equated to a “loss of face” for the PMI and the family. Hence, the stigma towards mental illness among Singaporeans- especially if a family member is a PMI - may manifest because of the need to preserve this “face” which is deeply intertwined with one’s social standing [21]. This postulation is reinforced by the findings from the previous local nationwide study, which revealed that Chinese Singaporeans as compared to Singaporeans of other ethnicities, reported the highest social distance scores towards PMI [20].

The attribution of mental illness to spiritual possession, while not unique to Singapore is an important belief that can influence stigma. Studies have found that superstitious beliefs in mental illness being a result of spiritual possession can evoke feelings of apprehension towards PMI [51]. Further, this misconception may also lead to help-seeking from inappropriate sources, delaying the PMI from receiving formal psychiatric treatment [52]. The attribution of mental illness to spiritual possession is the most apparent among the Muslims participants. Despite the drawback of such a belief, participants acknowledged that Muslims are the most tolerant and accepting towards PMI, though they did not explicitly elaborate on the reason behind it. A plausible explanation for this prosocial behavior towards PMI may be due to the Islamic belief that alienating the mentally ill equates to defying God’s will [28]. Moreover, the Qur’an and the Hadith (Islamic religious books) not only promulgate disabilities as something natural, but also impart principles and guidance for caring for disabled people [53]. Alternatively, it could be that mental illness is sometimes regarded as a trial or test bestowed upon the individual by Allah to allow the atonement for one’s sins, and this belief may in turn promote positive acceptance towards PMI [30].

Our finding on stigma being harsher in Asian as compared Western societies is corroborated by Shamblaw et al. (2015) study, whereby it was reported that Asians were associated with greater stigma towards mental illness than Europeans, with conservative values being highlighted as one of the underlying factor for such a relationship [17]. Anecdotally, because of conservatism, Asians tend to avoid discussing subjects which make them uncomfortable, and mental illness happens to be one of such topics. This avoidance towards acknowledging and talking about mental illness is perhaps compounded by the fact that Asians associate mental illness with stigma, shame and loss of ‘face’ [54]. As such, mental illness becomes a highly shunned topic among Asians, resulting in a vicious cycle eventuating mental illness into a taboo among the Asian communities.

The reluctance to talk about mental illness among Asians could be due also to the greater emphasis on moral attributions of mental illness. Krendly and Pescosolido's (2020) work on global differences in stigma reported higher stigma and greater emphasis on moral attributions in Eastern countries as compared to Western countries [55]. This resonates with the findings from our study, where some participants remarked that it’s harder to talk about mental illness in Singapore as compared to Western countries because individuals are blamed for their symptoms. As one participant puts it, “We always kind of blame it on like “oh he’s not doing so well in school, so he’s kind of feeling down again.” So we don’t really talk about depression, so I think in that way, if anyone suffers from depression, we don’t realize that they are suffering from depression. We just that think oh life is hard, yea so culture definitely plays a part, except I think it’s very different from Western culture, it’s a lot easier to talk about this kind of things.”

A rather local determinant of stigma is perhaps the “elitism mindset among Singaporeans” which may be related to meritocracy. In an earlier study pertaining to the treatment gap in Singapore, Subramaniam et al. (2019) discussed how meritocracy is highly regarded in Singapore and that people with higher education face greater inertia towards seeking treatment in order to conform to societal expectations [56]. In Yang et al. [57] study which was guided by a framework of “what matters most”, it was highlighted that mental illness stigma was contingent on the degree to which participants were able to achieve “what matters most” in their cultural context. As there is a strong emphasis on meritocracy in Singapore, stigma towards mental illness may arise due to the fact that many PMI have histories of disrupted academic or vocational careers, both of which happen to be highly valued in a meritocratic society. Furthermore, meritocracy facilitates negative evaluations and stereotyping towards ‘low status’ groups, as highlighted by a systematic review [58]. A plausible explanation for this is that because meritocratic worldviews are typically associated with the beliefs of a just world (i.e., I get what I deserve), it leads to a perception that PMI are responsible for their own illness and their inability to achieve in life [59].

The negative portrayal of PMI, such as attributing violence to mental illness in media reports [42] occurs in Singapore as well (see results), although in reality, PMI are more likely to become a victim of violent crime than be the perpetrator [60]. Nonetheless, media can be a useful tool to reduce stigma if used strategically [61]. In Singapore’s context, media could be used to help shift the stereotypes that the older generation have towards PMI. By portraying more characters with mental illness leading a meaningful life, instead of depicting PMI as violent/dangerous characters (see quote in results), media could potentially reduce the stigmatizing attitudes of the older generation, thus decreasing the likelihood of them inculcating stigmatizing views upon the younger generation.

Additionally, media could be a useful tool to destigmatize mental illness in Singapore, given that 78% of youths in Singapore use social media daily to access news and information, as reported by the National Youth Survey 2016 [62]. According to our findings, the taboo-ness associated with mental illness in Singapore is attributed to Asian conservative values. Hence, the utilization of social media platforms to disseminate factual information about mental illness can potentially alleviate this taboo-ness, for it exposes youths to mental illnesses and potentially fosters more conversations about mental illnesses as well.

Another strategy to reduce stigma in Singapore would be to educate individuals about mental health issues from young, thus enabling them to understand these conditions without being clouded by prejudice. This may also potentially disabuse youths of the stigmatizing views which may have been imparted by the older generation. A possible approach would be to introduce MHL modules in the school curriculum, an approach that has been considered superior to other school-based interventions [63]. Importantly, these MHL modules should also enlighten students that mental illness is not due to a personal defect or spiritual possession, but is instead a legitimate medical condition. Consequently, this may alleviate the concern that being associated with someone with mental illness or having a mental illness would result in a loss of “face” as mental illness will not be attributed to a moral defect on the sufferer’s part.

Our findings suggest that Singaporeans are likely to distance themselves from PMI due to several concerns, such as their perceived inability to handle the PMI or the perception that PMI are dangerous, and their unwillingness to work with PMI is due to the perception that PMI are burdensome. Anti-stigma campaigns in Singapore should, therefore, consider incorporating social contact with PMI as a component. Creating opportunities for social contact with PMI may help allay some of the aforementioned misconceptions, as well as reduce some of the intergroup bias that lay public hold towards PMI. The effectiveness of diminishing prejudice through contact has been supported by a meta-analysis of over 500 studies, which found that social contact can enhance knowledge regarding outgroup bias, increase empathy and perspective-taking [64]. A recent local study evaluated an intervention for college students comprising education and personal contact with a PMI (with a diagnosis of depression). The intervention was found to be efficacious in improving depression knowledge, reducing social distance and the stigma that persons with depression are dangerous/undesirable and weak-not-sick [65]. Such findings indicate that incorporating social contact in campaigns could be beneficial in assuaging the concerns of the lay public and thus reduce the unwillingness for social contact with PMI.

Lastly, campaigns in Singapore should also advocate that with proper treatment, PMI can hold down a job and lead successful/meaningful lives. This is especially important given the finding from our study which suggests that Singaporeans tend to be condescending towards those who seemingly lack the potential to become successful. Thus success stories are important to change this mindset. However, while campaigns can create cognitive shifts, the effects may be ephemeral [64]. Ultimately, to better reduce stigma, there may be a need to also implement legislative changes that promote inclusivity of PMI in workplaces and schools.

### Limitations

There are some limitations in our study that need to be acknowledged. As this study pertains to the topic of mental health stigma, the responses by participants may be affected by social desirability bias especially given the FGD setting. This was mitigated by the neutral manner of tone and words which the facilitator adopted in conducting the discussion, and the assurance at the start of the FGD that there are no right or wrong answers to any questions. Also, given the convenient sampling, there may be selection bias: participants who enrolled were individuals on two polarizing ends, namely those who have strong views against stigma towards mental illness and those with strong stigmatizing views towards people with mental illness. As such, our study’s results may not be generalizable to all the lay public in Singapore. Nonetheless, most of the reasons for stigma uncovered in this study largely concurred with those in existing literature, suggesting that selection bias was minimal.

## Conclusion

This study elucidated several determinants of stigma among the general public in Singapore using a qualitative approach. A total of 11 themes emerged, and these were classified into a socioecological model to illustrate how stigma is influenced by culture and environment. Findings from this study suggest that certain factors which cause stigma are quite pervasive across different cultures, such as the fear towards PMI, and the perception that PMI are burdensome. The determinants of stigma identified which were more culturally-specific were related to the Chinese concept of “face”, beliefs in spiritual possession, and Asian conservative values. The determinants of stigma that surfaced in our study which were unique were the elitist mindset among Singaporeans and the perceived inability to handle interactions with PMI. Lastly, findings of this study, must be incorporated into future anti-stigma campaigns in Singapore to ensure cultural misgivings and beliefs are addressed adequately.

## Supplementary information

**Additional file 1.** Topic guide questions pertaining to mental illness stigma and vignette.

## Data Availability

Readers who wish to gain access to the data can write to the senior author MS @ mythily@imh.com.sg to request access.

## Abbreviations

DALY: Diasbility-adjusted life years
FGD: Focused group discussions
GTHT: Gregory Tee Hng Tan
CMJG: Chong Min Janrius Goh
MS: Mythily Subramaniam
PMI: Persons with mental illness
SEMS: Socioecological explanatory model for stigma
SS: Shazana Shahwan
WJO: Wei Jie Ong
