# Supplementary material for: Mental illness stigma’s reasons and determinants (MISReaD) among Singapore’s lay public – a qualitative inquiry
Source: BMC Psychiatry. 2020 Aug 26;20:422. doi: 10.1186/s12888-020-02823-6 (PMC7448972; doi:10.1186/s12888-020-02823-6)
Supplement: Supplementary file 1 — Additional file 1. Topic guide questions pertaining to mental illness stigma and vignette. [file 12888_2020_2823_MOESM1_ESM.pdf]

Supplementary File 1: Topic guide questions pertaining to mental illness stigma and vignette

Thoughts about Mental Illness and Stigma

- “Can you describe a PMI? What comes to mind?”
- “Can you tell me more about such (repeat terms participants used to reflect stigmatizing attitude to first question if any) that you or other people have towards mental illness?”

Vignette

- The vignette below describes someone with **depression**

Adam is 30 years old. He has been feeling unusually sad and miserable for the last three weeks. Friends noticed he is no longer his usual cheerful self and he has declined all social gatherings over the past two weeks. Even though he is tired all the time, he has trouble sleeping almost every night. Adam doesn't feel like eating and has lost weight. He can't focus on his work and puts off making decisions. Adam feels worthless and even everyday tasks seem too much for him. This has come to the attention of his boss, who is concerned about Adam's poor work performance.

- The vignette below describes someone with schizophrenia

Mr Tan is 44 years old. He is staying in a 1-room HDB rental flat. He has not worked for years. He wears the same clothes every day and has left his hair to grow long and untidy. He is always on his own and is often seen sitting in the park talking to himself. Sometimes he stands and moves his hands as if to communicate to someone in nearby trees. He rarely drinks alcohol. At times he accuses shopkeepers of giving information about him to other people. He has put extra locks on his door. He says spies are watching him all the time. His neighbors complain that he does not clean his room which is becoming increasingly dirty and is filled with glass objects. Mr Tan says he is using these "to receive messages from space".

Follow-up questions on vignette (30mins)

- What, according to you, would people think about this person?
- Can you describe some of the positive or negative perceptions they might have?
- Do you think people will be willing to work with the person described in the vignette?
- Do you think people will be willing to include this person in their social or friend circle?

Role of culture in stigma

- Some people believe that the culture of the society plays a role in stigma. Would you agree with that? Can you tell us why you think that way?
